# “I would have to sell things in order to get the money”: A qualitative exploration of willingness to pay for the RTS,S/AS01 malaria vaccine in the Volta region, Ghana

**DOI:** 10.1371/journal.pone.0268009

**Published:** 2022-06-08

**Authors:** Sharon Darkwa, Gilles de Wildt, Maxwell Dalaba, Edem Vidzro, Evelyn Korkor Ansah

**Affiliations:** 1 College of Medical and Dental Sciences, University of Birmingham, Birmingham, United Kingdom; 2 Centre for Non Communicable Diseases Research, Institute of Health Research, University of Health and Allied Sciences, Ho, Ghana; 3 Centre for Malaria Research, Institute of Health Research, University of Health and Allied Sciences, Ho, Ghana; Universitat Jaume I, SPAIN

## Abstract

**Background:**

Malaria morbidity and mortality remain a challenge in Ghana. A promising childhood vaccine is being piloted in Ghana, however with the loss of its low-income status, Ghana is losing associated donor co-funding. User fees have been considered an alternative financing method, so this study utilised qualitative methods and explored caregivers’ willingness to pay for the malaria vaccine (RTS,S/AS01) to inform future service provision.

**Methods:**

The study design was cross-sectional. Twenty in-depth interviews were conducted between February 2020 and March 2020 amongst a purposive sample of caregivers of RTS,S/AS01 eligible children, in the Volta region, Ghana. Interviews were audio-recorded and transcribed into English Language. Thematic analysis followed, using NVIVO12 to organise this data.

**Results:**

Caregivers could distinguish between RTS,S/AS01 and routine vaccines and were willing to pay median GH₵5 (US$0.94), interquartile range GH₵3.75–5 (US$0.71–0.94) per dose of RTS,S/AS01. The maximum amount participants were willing to pay per dose was GH₵10 (US$1.88), interquartile range GH₵6–10 (US$1.13–1.88). Caregivers mentioned that they would work more to cover this cost because they were happy with services rendered to them during the RTS,S/AS01 pilot phase, and preferred vaccines over vector control measures. The results suggest that a willingness to pay was based on beliefs that the vaccine is fully effective. Although no participant declared that they would be unwilling to pay hypothetical user fees, there were still widespread concerns about affordability, with the majority feeling that the government should be responsible to pay for RTS,S/AS01.

**Conclusions:**

Participants expressed a willingness to pay due to an appreciation of vaccines, shaped by personal experiences with immunisations and disease. Participants’ average income was lower than the national average, potentially affecting the perceived affordability of RTS,S/AS01. Because of the belief that RTS,S/AS01 is fully effective, caregivers may pay less attention to other preventative measures, thus unintentionally undermining malaria vector control.

## Introduction

Despite a continuing decline in malaria-related mortality [1], malaria remains a significant issue in sub-Saharan Africa. Ghana, with 3% of the world’s disease burden for malaria [1], is considered a high-burden country. To date, RTS,S/AS01 (which represents the vaccine’s composition), is the only malaria vaccine approved for use [2] and is reported to reduce malaria incidence in children aged 5–17 months by 39% after four doses. Where its cost-effectiveness and pragmatic considerations are adequately scrutinised in hyperendemic pilot countries such as Ghana [3], RTS,S/AS01 could contribute to ambitious malaria eradication goals [4], particularly in under-fives, disproportionately affected because of a lack of acquired immunity [5].

In Ghana, childhood vaccinations are provided free at the point of access. The Global Alliance for Vaccines and Immunisations (GAVI), amongst other partners, has supported the Ghana Ministry of Health in co-financing the Expanded Programme on Immunization (EPI) [6, 7]. The World Bank now defines Ghana as a middle-income country [8]. Such countries graduate from GAVI’s financial support [9, 10] once a Gross National Income greater than US$1500 per capita is reached [11]. Therefore, Ghana is expected to graduate from GAVI’s financial support scheme by 2030 [6, 12]. Having previously defaulted on vaccine co-financing obligations [6], Ghana must find sustainable ways to finance the EPI and maximise coverage.

The RTS,S/AS01 pilot scheme is being delivered free to users, due to funding from non-governmental organisations [3]. If the pilot phase is successful, the vaccine may be integrated into Ghana’s EPI schedule. Therefore, similar strategies to finance its delivery in the long term must be sought. One method of achieving this could be through the introduction of user fees [13]. To establish the feasibility of this, Contingent Valuation—a method of service valuation where participants declare a willingness to pay (WTP) for a good or service [14]—can be used.

Quantitative WTP literature found that in Nigeria, participants were willing to pay US$5.06-US$6.77 for hypothetical malaria vaccines [15] and in Burkina Faso, US$2.42-US$3.54 [16]. Additionally, a higher WTP was associated with male sex, income, number of children and money spent on previous malaria treatment. These studies were conducted over a decade ago using the general public as their population group. Therefore, studying the WTP of caregivers involved in the ongoing RTS,S/AS01 pilot may enhance existing literature, as this population has a better awareness of the malaria vaccine it is being asked to value [17], and this study takes into account the current financial climate. Moreover, these earlier studies were based on hypothetical vaccines with no known literature on WTP for the RTS,S/AS01 malaria vaccine.

Despite limited literature on WTP for malaria vaccines, a review of WTP literature for immunisations against other communicable diseases in low and middle-income countries found a WTP for vaccines against Ebola [18] and dengue fever [19]. In the former, the rationale stemmed from a perceived lack of control over the spread of the disease. A systematic review [20] found a higher WTP for immunisation against chronic diseases, or those with higher morbidity and mortality, compared to acute disease. Malaria is generally an acute illness [21], so it would be useful to find out whether WTP for RTS,S/AS01 would follow the same pattern.

Additionally, determinants of WTP for vaccines appear to be diverse. The trend between spending on alternative preventative methods and WTP for vaccines for communicable diseases is not always consistent, both positive [15] and negative [19] trends have been found. Studies in the Philippines [22] and Indonesia [23], found that a history and good knowledge of the disease within the family, and use of alternative preventative methods predicted a higher WTP. Similar findings may be found for RTS,S/AS01, however there is no known literature allowing us to make this conclusion. The Ebola outbreak was an epidemic, so perceptions of severity as explored by Ughasoro et al [18] are likely to differ from an endemic disease such as malaria, in turn impacting their WTP. In addition, though dengue fever is also an endemic, mosquito borne disease, these studies [19, 22, 23] were conducted in Asia, where perceptions on endemic disease might differ from sub-Saharan Africa.

Research shows that qualitative WTP methodology improves the richness of quantitative WTP data, as it explores the rationale which led participants to choose to pay a particular amount [24, 25]. Although often absent from Contingent Valuation [25], qualitative methodology ‘bridges the gap’ between logical economic theory, and the true breadth of the issue, as participants are encouraged to reflect aloud [26]. Literature has found that justification of WTP values can be diverse, and includes a ‘moral satisfaction’ gained by paying their suggested amount, and a moral obligation to pay [24]. This suggests that the WTP figure alone is just one aspect of much richer data [24]. Through studying the acceptability of payments amongst caregivers of children given the vaccine, the feasibility of introducing service costs can be established. This can inform decision-making around service costs for vaccinations, as Ghana progresses towards cessation of GAVI support for the EPI [6]. This is especially relevant if the RTS,S/AS01 pilot is successful, and expanded nationwide. No such study has been conducted based on RTS,S/AS01, and to the best of our knowledge, this may be the first WTP study on this vaccine in Ghana.

Exploration of WTP for routine vaccines in addition to RTS,S/AS01 is necessary because although existing literature has shown that there is a general awareness of the role of vaccinations in sub-Saharan Africa [27, 28], in Ghana, many could not distinguish between the vaccines that their children had received [29]. RTS,S/AS01 could also be seen as a proxy for childhood vaccinations, as the EPI financing shift affects both emerging and existing vaccines [30].

This study aimed to qualitatively explore participants’ WTP for RTS,S/AS01, compared to routine childhood vaccines (S1 Table), and the factors influencing their decisions. This study was based on a hypothetical scenario if, following GAVI graduation, these vaccines were no longer provided free by donors or the government, with the aim of informing future service provision.

## Methods

### Setting

Data was collected in Akatsi North district—an RTS,S/AS01 pilot district in the Volta region of Ghana. It is one of 18 Municipalities and Districts in the region, with a population of over 33,000 and a population peak in the age range of 0–4 years [31]. Sharing a border with Togo, the district is entirely rural, with a major agricultural sector driving development [32]. Interviews were conducted in Ave Dakpa, the administrative capital of the district.

Over recent years, annual incidence of uncomplicated malaria in Akatsi North has increased, from 7681 cases in 2016 to 8411 in 2018 [33] despite a regional reduction. In May 2019, the RTS,S/AS01 pilot began in this district, targeting 139 children, so selecting this district was purposive. A single community was selected as this was an exploratory study: from our review of the literature, there was no known published data on the topic in English or with English language abstracts.

### Sample

The study population were primary caregivers of children aged 6–24 months, corresponding to the ages of children on the EPI schedule/receiving RTS,S/AS01. Participants were included where their child had received at least one RTS,S/AS01 dose, where the caregiver was able to give either written informed consent or indicate willingness to participate by use of thumbprint, and intending to remain in the study area throughout the study duration. Participants below the age of 18 were excluded.

### Sampling

Caregivers were recruited purposively during Child Welfare Clinics at Ave Dakpa health centre and Kpeduhoe Community-Based Health Planning and Services compound. These facilities were chosen because they were the only health facilities in the Ave Dakpa community, and Child Welfare Clinics are where mothers bring their children to receive both routine and RTS,S/AS01 vaccinations. Caregivers were approached to obtain those at different stages of the EPI schedule, including vaccine defaulters. They were then selected to obtain a variety of characteristics.

Community Health Nurses directed the research team to the residence of the eligible participants. Participants were approached in person by the research team. After being given a short description of the purpose and aims of the study, they provided informed consent. Two participants chose not to take part due to time constraints.

### Data collection

In-depth interviews (IDIs) were conducted based on a topic guide (S2 Table) between February and March 2020. This guide was developed based on WTP literature for vaccinations [15, 16, 34], applied guidance for Contingent Valuation qualitative studies [17] and study objectives. It was further refined following presentation to health researchers at the Institute of Health Research, University of Health and Allied Sciences (UHAS), Ghana and following pretesting. Pretesting was carried out in the same community with both a high income, and an unemployed individual, who also met the inclusion and exclusion criteria. One interview was conducted in English and the other in local language (Ewe), so that the topic guide could be developed for interviews to be conducted in either language. It was particularly evident that, as for Menaca et al [29], these participants could not distinguish between the routine vaccines on the EPI schedule, so the topic guide compared WTP for RTS,S/AS01 to all of the vaccines in the routine EPI schedule rather than just one specific vaccine.

Three research assistants (two male, one female) were trained to assist with the data collection. Interviews were conducted in either the local language (Ewe) or English by research assistants fluent in both. Of the 20 IDIs, 16 interviews were conducted in Ewe, three in Twi, and one in English.

The data collectors (research assistants) were graduate staff of the Institute of Health Research, UHAS. They were all involved in several studies including large-scale clinical trials, and were well-versed in the collection of all types of data. They had also been involved in the qualitative evaluation of the RTS,S/AS01 pilot implementation in Ghana. These research assistants were trained, based on the aims of this study and the topic guide.

Interviews were conducted in convenient, quiet locations in the community with only a research assistant, PI and caregiver present to ensure privacy. This was often outdoors, near the participants’ home. Conducting interviews in health facilities was thought to cause hesitancy in participant responses, and the research team wanted to disassociate themselves from the health facilities.

Before interviews began, participants provided informed consent and were asked questions from a demographics questionnaire, which was subsequently completed by the interviewer. This was developed using the Ghana Demographic and Health survey [35], and Macarthur Scale of Subjective Social Status (SSS) [36]. The latter is used where participants do not know, or do not want to report income and social status. To elicit SSS, participants ranked their perceived social standing on a ladder of ten rungs (S1 File). Rung one represented the lowest social status, whilst rung ten represented the highest. Interviews, lasting approximately 30 minutes, were audio-recorded onto encrypted devices.

### Analysis

To enhance translation accuracy, the same research assistants who conducted interviews carried out transcription and translation. Additionally, two transcripts at random were transcribed and translated into English by a second transcriber; these were compared to the original transcription to ensure the meaning was not lost during translation. Analysis was guided by Braun and Clarke’s ‘Phases of Thematic Analysis’ [37, 38]. Due to limited qualitative literature on this topic, an inductive approach was deemed most appropriate. Using NVIVO12 software, codes were systematically generated, and then second-coded by another researcher to reduce bias [39]. The two coded transcripts were compared for similarity. Similar codes were categorised into five main themes, derived from the data. Constant comparison was necessary to ensure that saturation of themes was reached, so interviews were carried out iteratively.

Quantitative WTP data was collated and reported as median and IQR as the data set was skewed. All costs were collected in Ghana cedis (GH₵) and results presented in both GH₵ and US$ using the average exchange rate of 2020 (1US$ = GH₵5.3).

### Ethical considerations

Ethical approval was obtained from the University of Birmingham Internal Research Ethics Committee and UHAS Research Ethics Committee in Ho, Ghana. In addition, permission was sought from the District and Regional Health Directorates of the study site. Before each interview, written informed consent, or thumbprint indicating willingness to participate, was obtained from participants. Confidentiality and anonymity were maintained throughout data collection and analysis.

## Results

### Background characteristics of caregivers

Table 1 presents the demographic characteristics of study participants (caregivers). Despite criteria inclusive of both males and females, primary caregivers were all female. Most caregivers were between 21–40 years old and half had between one and two children. At the time of interview, most children had received 3 doses of the RTS,S/AS01. Half of the participants’ children had received their last RTS,S/AS01 dose less than two months prior to interview, and half of the participants reported having a family member who had previously had malaria. The average monthly income of caregivers was GH₵300/US$57 and below. Fifty-five percent of the caregivers’ Subjective Social Status (SSS) was ranked greater than five.

**Table 1:** Participant demographics.

Variable	N (%)
	[N = 20]
Age	
≤20	2(10)
21–30	8(40)
31–40	8(40)
41–50	2(10)
No. of children	
1–2	10(50)
3–4	5(25)
5–6	5(25)
RTS,S/AS01 doses received	
1	1(5)
2	7(35)
3	12(60)
Average time since last RTS,S/AS01 dose	
≤1month	10(50)
2 months	3(15)
3 months	4(20)
4 months	2(10)
5 months	0(0)
6 months	1(5)
Participant-reported family history of malaria	
Yes	10(50)
Monthly net income (GH₵)	
n/a	5(25)
≤300	10(50)
301–600	2(10)
601–900	0(0)
>900	3(15)
Subjective Social Status (SSS) (S1 File)	
≤5	9(45)
>5	11(55)

Five main themes were identified from the data, under two broad categories: WTP for RTS,S/AS01, and the various factors influencing this willingness. This has been summarised in Table 2, and is discussed below.

**Table 2:** Summary of themes.

Objective	Theme
RTS,S/AS01 WTP	Comparing WTP for RTS,S/AS01 and routine vaccines (S1 Table)
Factors influencing WTP	An appreciation of services
	The wellbeing of the child
	The ability to pay
	The influence of vector control

### Comparing WTP for RTS,S/AS01 and routine vaccines

All caregivers were aware that their child was involved in the RTS,S/AS01 pilot programme; one caregiver (F10) even identified an RTS,S/AS01 sticker placed on her child’s health record book. Comparison of WTP between RTS,S/AS01 and routine vaccines highlighted a fairly even division between those who were willing to pay more for RTS,S/AS01, and those willing to pay more for routine vaccines. Of those willing to pay more per dose of RTS,S/AS01 over routine vaccines, costs were predicted to be higher, and malaria was considered a more important disease to protect against. When asked to justify a higher WTP for RTS,S/AS01 over routine vaccines, disease severity and prevalence were considered:

*“Malaria is a very serious sickness […] If you’ve ever had malaria you will understand […] So, the fee for [RTS,S/AS01] is likely to be high.”*

*(IDI-F10, SSS = 3)*

*“In this community it is mostly malaria that we suffer from.”*

*(IDI-F16, SSS = 6)*

Of those willing to pay more per dose for routine vaccines over RTS,S/AS01, the most common justification was rather that they did not feel that RTS,S/AS01 was as costly as routine vaccines.

*“Not all vaccines cost the same in my opinion … I don’t expect the malaria vaccine to be very expensive.”*

*(IDI-F07*, *SSS = 4)*

Amongst this group, the efficacy of RTS,S/AS01 was also a contributing factor for WTP. For instance, a participant mentioned that the partial efficacy of RTS,S/AS01 was a reason for her WTP being less for RTS,S/AS01 compared to other vaccines:

*“Those vaccines protect children better than the malaria vaccine”*.

*(IDI-F15, SSS = 6)*

Another participant assigned a lower price to RTS,S/AS01 to allow for the purchase of other malaria preventive methods:

*“Some people may decide to use the money to purchase other methods of protection such as mosquito coils”*.

*(IDI-F13, SSS = 5)*

Few study participants said there was no difference in how much they would be willing to pay for RTS,S/AS01, and routine vaccines. However, a lack of financial means was the most common reason mentioned.

***Interviewer**: “What you mentioned [GH₵5] is just the same for other vaccines. Why?”*

***Participant**: “Yes it’s because there is no money, that’s why I mentioned that.”*

*(IDI-F01, SSS = 1)*

However, some caregivers said there would be no difference in WTP between RTS,S/AS01 and routine vaccines, given that all vaccines were the same.

*“It wouldn’t be different because they are all the same vaccine.”*

*(IDI-F14, SSS = 8)*

It was revealed that for a single RTS,S/AS01 dose, participants were willing to pay a median of GH₵5 (US$0.94), interquartile range (IQR) GH₵3.75–5 (US$0.71–0.94). The maximum amount participants were willing to pay per dose was GH₵10 (US$1.88), IQR GH₵6–10 (US$1.13–1.88). Though participants expressed concern that other caregivers in the community may be unwilling to pay if user fees were introduced, no study participant declared that they themselves would be unwilling to pay for RTS,S/AS01 regardless of its price. WTP did not appear to have much variation with income, however caregivers generally said that SSS was taken into consideration when deciding their WTP.

*“It [SSS] will surely influence how much I’m willing to pay because if I were on the lowest level [of the ladder], I may not mention any amount at all.”*

*(IDI-F07, SSS = 4)*

### An appreciation of services

Caregivers were able to consider the benefits of free vaccinations on a larger scale, feeling that their contributions would be worthwhile.

*“I think payment for the vaccines will reduce the pressure on the government… because the population has increased now.”*

*(IDI-F06, SSS = 10)*

Participants gave a wide range of suggestions of what their payment could go towards, referring to the maintenance of stock, vaccine production, and health worker salaries, as an appreciation for their hard work. Having adequate funding to expand the RTS,S/AS01 pilot was also important to some participants—a caregiver mentioned that she had defaulted on RTS,S/AS01 because she had travelled to a non-implementing region. Therefore, expansion of the current pilot was important to her so that her child could continue to benefit from the service regardless of location.

*“I travelled to Tarkwa, which was not one of the districts implementing the pilot programme so I had to continue when I came back […] I think the payment will make it possible for the services to be extended to other areas as well.”*

*(IDI-F20, SSS = 5)*

Though study participants mentioned their various WTP for RTS,S/AS01, a minority felt that the amount they suggested did not adequately match the service that was being provided to them.

*“…expenditure is huge […] even though the vaccines may cost more than that, I know that they sometimes reduce the costs or make it free for the sake of good health for our children.”*

*(IDI-F20, SSS = 5)*

The advantages of charges or free services for the vaccination were raised given that it would promote utilisation. Some study participants were of the view that health services that are provided free of charge to community members are usually not well appreciated or valued. They therefore suggested that payments for vaccinations would improve the attitudes of both caregivers and health workers in the provision of childhood vaccine services.

*“Anything that is free… they don’t really pay much attention to it […] But when you pay, that’s where they value it […]. They are […] toiling for it, [so] would rather come. That is what I see with some community members.”*

*(IDI-F14, SSS = 8)*

*“It will make people take the vaccines seriously including the health providers since they know it’s being paid for and because the nurses travel long distances to some communities […] the fact that the service is free will not motivate them to do more.”*

*(IDI-F07, SSS = 4)*

*“There will be advantages because the nurses would be excited knowing that we are paying for it.”*

*(IDI-F16, SSS = 6)*

### The wellbeing of the child

The wellbeing of the child was the most common factor influencing WTP. The majority of the caregivers said that their child being protected from vaccine-preventable diseases was most important to them when deciding their WTP, since their child “wouldn’t be able to say if he is sick” (F16). WTP to make their child ‘healthy’ (F04, F13) was shaped by positive experiences with the vaccine. Limited adverse events reinforced their belief about the safety of RTS,S/AS01.

***Interviewer**: “What would make you vaccinate your child even if you have to pay?”*

***Participant**: “My child has never been sick ever since he started receiving the vaccinations so I can testify of how good the vaccine is, and that is why I will still vaccinate my child.”*

*(IDI-F06, SSS = 10)*

Some caregivers showed implicit understanding that receiving less than four doses does not provide full immunity, citing that they should “continue sleeping under bed nets even after taking the malaria vaccine” (F12), and that children “shouldn’t be left for mosquitoes to bite them” (F19). At this stage of the pilot, children had received at most, three doses of a four-dose regimen, and only after the fourth dose is immunity thought to be conferred [2]. However, the vast majority of the caregivers felt the vaccine was already working.

*“It works […] after receiving the vaccine I give her a cold bath then that’s it, it works […] she hasn’t had malaria.”*

*(IDI-F01, SSS = 1)*

Trust in healthcare professionals also reinforced participants’ belief about RTS,S/AS01’s safety. They were seen as ‘experts’ (F10) and important in debunking misinformation.

*“Some people heard on the radio that when a child was vaccinated with the malaria vaccine the child died … so when they said they will give the malaria vaccine I refused. The nurses explained that it wouldn’t harm the child before I allowed my child to be vaccinated with it.”*

*(IDI-F03, SSS = 10)*

Though a caregiver had a bad experience with the service provided to her, she still said she would be willing to pay to vaccinate her child “so that the child would remain safe”, but would expect communication with healthcare professionals to improve:

*“They fail to focus on you […] The child is sick and […] as such, I am becoming the doctor doing the diagnosis myself. This is because they did not ask me any question like, this week, how has your child been? […] Not all of us know much. They should be patient with us.”*

*(IDI-F18, SSS = 2)*

Ultimately, there was a consensus that paying for an effective vaccine (as RTS,S/AS01 was seen to be) would be a far smaller burden than costs such as “paying for laboratory costs” (F16) and the inconvenience (F04, F06, F09, F14, F19) of treating malaria should the child get the disease.

*“I think it’s a great help you are giving us the parents. Because if a child is sick and you send them to the clinic you would pay a lot of money so I would prefer to pay this to prevent my child from getting sick.”*

*(IDI-F01, SSS = 1)*

There was evidence that study participants have similar views and experiences about other routine vaccines with regards to treatment costs and vaccines:

*“When I had my first child, I was diagnosed with Hepatitis B and was asked to pay GH₵850 for my child to be given an injection […] the important factor is the protection the vaccine offers to my child because I think it will cost me more to treat a disease than to prevent it through vaccination.”*

*(IDI-F07, SSS = 4)*

### The ability to pay

The ability to pay for RTS,S/AS01 was also explored, and it was revealed that ability to pay was the second most common factor influencing WTP (after the wellbeing of the child). Some caregivers said that their suggested amounts would be readily available, saying GH₵5 was ‘inexpensive’ (F17), and GH₵8, ‘wouldn’t be a burden’ (F14) due to effective budgeting. However, the majority of caregivers said they would need to work more to be able to pay, from businesses such as trading (F13, F20), as a seamstress (F18), and taking up additional labourer jobs (F03, F05).

*“I would have to sell things to get the money […] we would have to work extra hard […] Through my work and strength I know I will be able to pay for it.”*

*(IDI-F16, SSS = 6)*

Some study participants mentioned that they have fluctuating income and so though they may be able to afford it now at their suggested price, their ability to mobilise finances was unpredictable:

*“Some years the farming is good, we get money, but in other years we don’t get anything.”*

*(IDI-F02, SSS = 6)*

A caregiver, who’s Subjective Social Status was 3, felt that her social status was subject to change, and this would limit her ability to pay for vaccinations if this ever became a requirement.

*“I might not be able to afford it always […] Looking at my twins it means if I’m not willing to borrow, I will wait until I have the money before the children will get vaccinated.”*

*(IDI-F10, SSS = 3)*

Moreover, her situation was unique as a mother of twins and six children in total, however she felt it was her duty as a parent to find the means–something she was aware of before she had children:

*“The moment you are married proves that you’re ready to have children so you must prepare for the expenses that come with that.”*

*(IDI-F10, SSS = 3)*

Despite making few decisions regarding the health of the child, many caregivers felt that the father should take some responsibility should user fees be introduced. The majority of caregivers said paying would primarily be the responsibility of the child’s father, and some further mentioned that they would not send the child for the vaccination if the father does not pay. There was also evidence of shared financial decision-making. However, in many households, the father was not around, sometimes working in other regions, so their contribution was sometimes limited:

*“Myself and my husband [would pay] […] My husband is not with me here, he’s in the Upper West region […] I’ll just contact him, then he sends a token to us. When I get to the health facility [..] maybe the amount he has sent is not enough […] so, I will top up.”*

*(IDI-F14, SSS = 8)*

Though most caregivers said they would continue to vaccinate if user fees were introduced, these individuals also considered the affordability from the perspective of those within their community, feeling that “all hands are not equal” (F16) and that there would be people who couldn’t afford it at any price:

*“Some people will refuse to send their children for vaccinations because of the payment […] people who are not working and […] single parents because the father of the child refused to accept responsibility […] for some of them, the grandparents of the child are the ones paying for the upkeep of the child […] it will be a burden.”*

*(IDI-F06, SSS = 10)*

A minority (F06, F17, F18) said that they themselves may not vaccinate their child if user fees were introduced. Most caregivers felt that it should be the government’s responsibility to pay.

*“They [government] want the child’s safety, so it’s better they provide…they say they are our future leaders so…”*

*(IDI-F09, SSS = 6)*

Some study participants suggested ways to make it easier for households to pay. One such suggestions was combining RTS,S/AS01 with other immunisations, as was done for the pentavalent vaccine. They explained that this would reduce the number of vaccines given to children overall, feeling that the number of vaccines currently given to children is so many. Participants further mentioned that the combining vaccines may result in them working better (F10, F15), thus boosting the immunity of the child (F10).

*“You can merge some vaccines together to reduce the number […] the cost will come down for parents.”*

*(IDI-F14, SSS = 8)*

Another participant raised the idea of cross-subsidisation–higher-income households could pay to allow lower-income families to access RTS,S/AS01 for free.

*“If they began charging for the vaccinations, they could probably use that money to assist those who can’t afford it at all.”*

*(IDI-F05, SSS = 10)*

### Use of other malaria prevention methods

The majority of the study participants acknowledged that insecticide-treated nets (ITNs) was a good form of malaria prevention and the majority mentioned that they own at least one. However, WTP for RTS,S/AS01 was not greatly influenced by use of other malaria prevention methods, and some caregivers mentioned that if a child receives RTS,S/AS01, there would no longer be a need for ITNs. Caregivers expressed a preference for RTS,S/AS01 because ITNs trap heat, making it uncomfortable for children to sleep under them. They also revealed that due to the low durability of the nets, the nets easily get torn, allowing mosquitos to pass through to bite the children. Furthermore, given that people are generally mobile and cannot carry ITNs everywhere they go, they preferred RTS,S/AS01 to the ITNs.

*“If you go somewhere and you don’t have access to a bed net, because the child has been injected with the malaria vaccine, when mosquitoes bite the child, [they] will not get serious malaria […] So, I will say the net is good but the vaccine is better.”*

*(IDI-F20, SSS = 5)*

Caregivers had various reasons for preference of RTS,S/AS01 to other malaria prevention methods too, such as the use of mosquito coils which caregivers frequently linked to the onset of respiratory problems.

Generally, when caregivers were asked to justify their preference for the vaccine, the main reasoning was a belief that RTS,S/AS01 was more effective, as it “works from within” (F01, F03, F07, F10, F19, F20).

*“Other preventive methods are just temporary and so once they’re not in place you can be at risk. But for the vaccine, it is in the body and working all the time.”*

*(IDI-F07, SSS = 4)*

*“If you receive the vaccine, you will not get Malaria again.”*

*(IDI-F18, SSS = 2)*

Notwithstanding the support of RTS,S/AS01 over ITNs by the majority of the study participants, a few caregivers did express a preference for other malaria prevention methods. This preference was not based on the efficacy of RTS,S/AS01, and justifications were often unclear.

*“I will choose the bed nets over the malaria vaccine [but] even though we’re sleeping under bed nets, when we go to the health facility, they say that we have malaria which is what I don’t understand.”*

*(IDI-F15, SSS = 6)*

Because caregivers generally preferred vaccines to other malaria control methods, caregivers indicated that they would pay to ensure their child would be protected in a way that other malaria control methods could not.

***Interviewer:** “You already have two nets. If you didn’t, would you mention a different amount?”*

***Participant:** “No, it doesn’t have to change […] because, it is not always that we are in a net. When we are outside, mosquitoes can bite us.”*

*(IDI-F18, SSS = 2, 2ITNs)*

Misuse of ITNs was mentioned, and this was because they had been provided to them for free by the government.

*“They have been giving us mosquito nets for long, so we pay less attention to it.”*

*(IDI-F19, SSS = 3)*

*“The last time I went to Kpeduhoe, someone had used the mosquito nets […] to cover this plant.”*

*(IDI-F14, SSS = 8)*

As a result of this, some caregivers suggested that vaccination user fees may therefore be beneficial.

*“The government has made provisions for mosquito nets, and we are not sleeping in it because of the heat, and we want to have this one free, government will run at a loss.”*

*(IDI-F10, SSS 3)*

However, this was not to dismiss the role of other malaria prevention methods. Caregivers still found it necessary, as a caregiver (F09) who initially scored her Subjective Social Status as 6 said *“If I were [at SSS] number 2 and can no longer afford [RTS*,*S/AS01] I would still stick to the mosquito nets”*. If she could no longer afford the vaccine, the free ITNs would suffice in protecting her child.

## Discussion

### WTP

The study explored caregivers’ willingness to pay for the new malaria vaccine, “RTS,S/AS01” in the Volta region of Ghana.

RTS,S/AS01 could be seen as a proxy for childhood vaccinations, as the EPI financing shift affects both emerging and existing vaccines [30]. However, caregivers could distinguish between RTS,S/AS01 and routine vaccinations. Some caregivers felt that RTS,S/AS01 would be cheaper, whilst others saw malaria as a more serious disease in their community, so would pay more. The latter suggested that user fees for routine vaccines may be less well received, as the diseases they protect against are rarer.

Ghana is in early stages of GAVI transition [40] so is still receiving donor financial support such that childhood vaccinations are still provided for free [6]. Typically, reduced donor financial support in middle-income countries reduces vaccine coverage and consequently, there are often resultant rises in preventable deaths [9]. This has already been previously observed in Ghana, where a reduced health budget was associated with a 10% reduction in pentavalent vaccine coverage [6]. This study demonstrates that caregivers may well still receive, and be willing to financially contribute in the payment of childhood vaccines if GAVI support was no longer possible. However, this may be a burden to households given that participants’ average gross monthly income was US$57/GH₵300 or compared to US$84/GH₵446 [41] nationally per capita.

Participants chose values which were affordable to them. Some also perceived that RTS,S/AS01 delivery was less expensive than the delivery of each of the routine vaccines. In these stages of RTS,S/AS01 procurement, expenses cannot be fully established, however for routine vaccines, the estimated cost to the government/ funding bodies of a fully immunised child in Ghana is US$60 [6], averaging at around $3.16 per dose for the routine EPI schedule (based on an assumption that all routine vaccines, including combination vaccines, were of equal cost) (S1 Table). This is more expensive than even the maximum amount participants were willing to pay per RTS,S/AS01 dose (US$1.88). Additionally, though GAVI-supported countries are protected by lower supply agreement prices during their transition from financial support [42], these prices are rarely sustained in the long-term [30] and the national cost to acquire these vaccines could rise further in the future. Therefore, should the pilot be expanded without subsidy, relying solely on user-fees may put undue burden on caregivers.

Contributions from citizens were suggested as a sustainable financing method in other countries such as Angola and Bhutan [43, 44]. These countries, who no longer receive financial assistance from GAVI, outlined and implemented plans for people to contribute towards national health trust funds. Nevertheless, in Bhutan, contributions from the working population comprised just 1% of the total fund [45]. These examples are not entirely comparable to this study in Ghana, where user-fees were hypothetically introduced at the point of care rather than into a national fund. However, the amounts that this study’s participants were willing to pay (US$0.94/GH₵5) may not be noteworthy in ensuring sustainable financing of RTS,S/AS01, especially since many felt that these values did not amount to the true costs of RTS,S/AS01. This value is equivalent to a mosquito coil [46] in Ghana, and largely matches quantitative findings from Burkina Faso [16], (mean WTP US$1.91), though less so in Nigerian findings [15] (mean WTP US$5.06–6.77). However, it should be noted that conversion of our results using Purchasing Power Parity (PPP) rates [47], rather than formal exchange rates, yields a higher WTP (US$2.35).

Service costs and demand are inextricably linked [15, 16]. In China, introducing user fees for non-EPI immunisations resulted in a coverage of just 10%, despite a vaccine efficacy of 97% [16, 48]. In our study, caregivers acknowledged that other caregivers would stop vaccinating, though none of the participants themselves indicated an unwillingness to pay for vaccinations, contrasting previous quantitative study findings which was up to 14.5% [15].

### Influencing factors

The wellbeing of the child was a major WTP consideration. In this study, participants were generally not sceptical about the vaccine’s effectiveness; consequently, should the RTS,S/AS01 programme be expanded, it seems unlikely that this will act as a deterrent in caregivers’ WTP user fees. Additionally, the provision of routine vaccines for free appears to lead to a greater appreciation for the services rendered to them, and therefore appeared to be an influencing factor in WTP for RTS,S/AS01. Similar perceptions may exist nationally, provided that the EPI services remain free at the point of access.

Literature [15, 16] found that income was a strong WTP predictor however, income sources were not well established. Our study shows that fathers were important in financial decision-making, and quantitative studies suggested that males were usually willing to pay more than their female counterparts [16, 48]. However, in many cases, fathers were away from the family for extended periods and could therefore not always be relied upon for payment. Sources of income forming WTP rationale were therefore based on the primary caregiver alone, rather than the household. Caregivers reported fluctuating income, suggesting that their WTP was dependent on how much they could mobilise from their work at a given time. Therefore, there was an important distinction between a caregiver’s ability, and willingness to pay. Uptake of preventative interventions correlates with socioeconomic status [49], so without income-related support, user fees could make malaria a disease even more greatly affecting the poor. Ghana’s National Health Insurance Scheme was created with this equitable access in mind [50], however with increasing obligations [6], perhaps cross-subsidisation could be a feasible funding base.

Of those receptive to user fees, caregivers cited the cost and inconvenience of treating malaria, confirming Nigerian findings [15] that those who had previously been treated for malaria, specifically those who had paid more for treatment, were willing to pay more for a hypothetical malaria vaccine. There was an appreciation of the services offered to them so far for free, and participants felt that others in the community would consequently take these services more seriously.

In Nigeria, tolerable hypothetical malaria vaccines were preferred over efficacious ones [15]. Additionally, a systematic review by Dimala et al found that a fear of side-effects could act as a barrier to the receptivity of RTS,S/AS01 [51]. Caregivers in our study location were willing to pay because of the *absence* of serious adverse events since the child’s immunisations began. They also had the belief that RTS,S/AS01 was fully effective despite the child having received less than the full four doses, showing participants did not grasp the concept of RTS,S/AS01’s partial efficacy of 39%, conferred only after all four doses [2]. Even where there was an implicit understanding of this (through continued use of other malaria prevention methods), this was not considered when justifying their WTP. Results demonstrate a trade-off between affordability and the child’s wellbeing, so if user fees were introduced, and caregivers believed immunity was conferred at less than the full course, drop-out for the latter doses could become customary. This is especially important as RTS,S/AS01 coverage beyond the first dose is already an issue in this district, as was outlined in the regional review.

Literature found that ITNs were the malaria prevention method most widely used across Ghana [52]. However only 54% of households who owned ITNs used them, despite a target of 80% [53]. Our results explained that although all participants owned ITNs, they were not always used as they should have been (being free, they were sometimes taken for granted), and many considered them to be less effective than the vaccine. As a result, vector control methods did not influence their WTP. Caregivers were keen to take the responsibility of malaria prevention away from themselves. Bed nets have an efficacy (ideal world performance [54]) of 50–60% [55], but effectiveness (real-world performance) as low as 17% often because of incorrect use. Participants in our study saw more appeal in the mechanism of action of vaccines, working from inside the body.

These findings raise the risk that over-estimation of the protective effect of RTS,S/AS01 which in reality, is 39%, could lead to caregivers using the vector control methods less in future. A study conducted in Uganda found that there were higher odds of a bed net being correctly used where they were specifically sought (and purchased) by the household, rather than those that were provided for free [56]. Since 2015, the use of ITNs has not markedly progressed [1]. Future research could include studying the impacts of the expansion of RTS,S/AS01 on their uptake and use.

Caregivers were generally willing to pay if the payment of user fees was to become mandatory. They were, however, of the view that the government should be responsible for covering the cost of vaccination as they have done with all other childhood vaccines. Globally, this is common practice, even for countries with a higher GDP. Vaccines are often treated as a public good, due to greater positive externalities—countries find greater benefit than that conferred to the child alone [57]. Routine childhood immunisations are free in the UK [58] and to uninsured and underinsured children in the US [59]. It could be argued for RTS,S/AS01 that because malaria is not infectious, but instead spread by a vector, there is a shift away from government responsibility—achieving a high coverage does little to sustain herd immunity [60, 61]. However, malaria costs the Ghanaian economy around 6% of their GDP annually [62], so where political will is high, these needs may be met [63].

## Limitations

All our participants were female, because many fathers were not present at the times that the interviews were conducted. Of those that were present, they did not meet criteria as the primary caregiver of the RTS,S/AS01 eligible child and it was clear that mothers were more aware of vaccination services. Sauerborn et al [16] suggested that male WTP was higher than their female counterparts, but this could not be determined from our sample. Secondly, none of the participants declared an unwillingness to pay for RTS,S/AS01. However, they did indicate the possibility that “other members of the community” may be unwilling to pay. Two participants, despite reassurance, were hesitant to declare their WTP, because of concerns that they may end up being charged. For these reasons, the possibility that participants may not have been entirely upfront about their willingness to pay during interviews is therefore not inconceivable. Additionally, the sample was purposive, leaving the study open to the possibility of researcher bias. Participants in this study were from a rural agricultural area. These factors may restrict the generalisability of the findings of the study.

Some of the IDIs were conducted in the local languages of the study area and translated into English for analysis. It is possible that the actual meaning of some statements made in the local languages may have been lost in English translation. Nonetheless, the interviews were translated and transcribed by experienced research assistants who are fluent in both English and the local languages, hence minimizing the potential mistranslation.

## Conclusion

A WTP of GH₵5/US$0.94 per RTS,S/AS01 dose, in this rural community was largely influenced by an appreciation of the role of immunisations, though offset by affordability concerns both short and long-term. WTP was shaped by personal experiences with immunisations and disease. Though most would be willing to pay mandatory user fees, many felt that the government should be responsible for covering this cost, and not them. An overestimation of the protective effect of RTS,S/AS01 could impact uptake and use of vector control methods, and further research on this is recommended.

Despite the limited generalisability of qualitative literature, this study can be useful alongside ongoing Health Utilisation and costing studies, in the evaluation and decision-making for RTS,S/AS01. These findings are also important in informing national financial planning for immunisations. Further research could benefit from hearing the perspectives of the fathers of RTS,S/AS01 eligible children, particularly as head of household. Finally, research on how to increase vaccination coverage for the poor, where there are user fees, is recommended.

## Supporting information

S1 Table Ghana EPI schedule. (DOCX)

S2 Table Topic guide summary. (DOCX)

S1 File Demographics questionnaire and MacArthur scale of subjective social status [36]. (DOCX)

S2 File Inclusivity in global research. (DOCX)

## References

[1] World malaria report 2019. Geneva: World Health Organization; 2019. Licence: CC BY-NC-SA 3.0 IGO.

[2] RTS S. Efficacy and safety of RTS,S/AS01 malaria vaccine with or without a booster dose in infants and children in Africa: final results of a phase 3, individually randomised, controlled trial. The Lancet 2015;386(9988): 31–45. doi: 10.1016/S0140-6736(15)60721-8 25913272 PMC5626001

[3] Ministry of Health/Ghana Health Service. Introduction plans for RTS,S/AS01 Malaria Vaccine Implementation Programme. 2017.

[4] World Health Organisation. Global Technical Strategy for Malaria 2016–2030. 2015 [cited 2019 Nov 11]. Available from: https://apps.who.int/iris/bitstream/handle/10665/176712/9789241564991_eng.pdf;jsessionid=0676E8EC11F40A56A58024E1EF944B01?sequence=1.

[5] Snow R, Omumbo J. Chapter 14: Malaria. In: Jamison D, Feachem R, Makgoba MW ea, editors. Disease and mortality in Sub-Saharan Africa. 2nd ed. Washington (DC): Dev Brief / World Bank. 2006.

[6] Cashin C, Arias D, Bloom D. Immunization financing: a resource guide for advocates, policymakers, and program managers. Washington (DC): Results for Development 2017.

[7] Medecins Sans Frontieres. The Right Shot: bringing down barriers to affordable and adapted vaccines. Medecins Sans Frontieres 2015.

[8] The World Bank. World Bank Country and Lending Groups. 2010 [cited 2019 Nov 11]. Available from: https://datahelpdesk.worldbank.org/knowledgebase/articles/906519.

[9] World Health Organisation. The Middle Income Strategy. 2016 [cited 2019 Nov 11]. Available from: https://www.who.int/immunization/programmes_systems/sustainability/mic_strategy/en/.

[10] Force MCT. Sustainable Access to Vaccines in Middle-Income Countries (MICs): A Shared Partner Strategy. Report of the WHO-Convened MIC Task Force. World Health Organization 2015.

[11] Results For Development. Graduating countries from GAVI assistance. 2012 [cited 2020 Feb 18]. Available from: https://www.r4d.org/projects/graduating-countries-gavi-assistance/.

[12] Zakariah A. Statement by Ghana. no date [cited 2020 May 21]. Available from: https://apps.who.int/gb/Statements/WHA68/PDF/Committee%20A/Eleventh%20meeting/GHANA%20GLOBAL%20VACCINE%20ACTION%20PLAN.pdf.

[13] World Health Organization. Brief 5: User fees for immunisation. 2010 [cited 2020 Apr 22] Available from: http://origin.who.int/immunization/programmes_systems/financing/analyses/Brief_5_User_Fees.pdf.

[14] Alberini A, Cooper J. Applications of the contingent valuation method in developing countries: A survey.: Food & Agriculture Org.; 2000.

[15] Udezi WA, Usifoh CO, Ihimekpen OO. Willingness to pay for three hypothetical malaria vaccines in Nigeria. Clin Ther. 2010 Aug;32(8): 1533–1544. doi: 10.1016/j.clinthera.2010.07.018 20728765

[16] Sauerborn R, Gbangou A, Dong H, Przyborski JM, Lanzer M. Willingness to pay for hypothetical malaria vaccines in rural Burkina Faso. Scand J Public Health. 2005;33(2): 146–150. doi: 10.1080/14034940510005743 15823976

[17] Johnston RJ, Weaver TF, Smith LA, Swallow SK. Contingent valuation focus groups: insights from ethnographic interview techniques. Agric Resour Econ Rev. 1995;24(1): 56–69.

[18] Ughasoro MD, Esangbedo DO, Tagbo BN, Mejeha IC. Acceptability and willingness-to-pay for a hypothetical Ebola virus vaccine in Nigeria. PLoS Negl Trop Dis. 2015;9(6): e0003838. doi: 10.1371/journal.pntd.0003838 26076007 PMC4467844

[19] Hadisoemarto PF, Castro MC. Public acceptance and willingness-to-pay for a future dengue vaccine: a community-based survey in Bandung, Indonesia. PLoS Negl Trop Dis. 2013;7(9): e2427. doi: 10.1371/journal.pntd.0002427 24069482 PMC3777870

[20] Kim S, Sagiraju H, Russell LB, Sinha A. Willingness-to-pay for vaccines in low-and middle-income countries: A systematic review. Annals of Vaccines and Immunization. 2014;1(1): 1001.

[21] World Health Organisation. International Travel and Health: Malaria. 2011 [cited 2020 Apr 1]. Available from: https://www.who.int/ith/diseases/malaria/en/.

[22] Palanca-Tan R. The demand for a dengue vaccine: a contingent valuation survey in Metro Manila. Vaccine. 2008;26(7): 914–923. doi: 10.1016/j.vaccine.2007.12.011 18206277

[23] Harapan H, Anwar S, Bustamam A, Radiansyah A, Angraini P, Fasli R, et al. Willingness to pay for a dengue vaccine and its associated determinants in Indonesia: a community-based, cross-sectional survey in Aceh. Acta Trop. 2017;166: 249–256. doi: 10.1016/j.actatropica.2016.11.035 27908746

[24] Schkade DA, Payne JW. How people respond to contingent valuation questions: a verbal protocol analysis of willingness to pay for an environmental regulation. J Environ Econ Manage. 1994;26(1): 88–109.

[25] Chilton SM, Hutchinson WG. Do focus groups contribute anything to the contingent valuation process? J Econ Psychol. 1999;20(4): 465–484.

[26] Borghi J, Shrestha DL, Shrestha D, Jan S. Using focus groups to develop contingent valuation scenarios—A case study of women’s groups in rural Nepal. Soc Sci Med. 2007;64(3): 531–542. doi: 10.1016/j.socscimed.2006.09.028 17107740

[27] Ojakaa DI, Ofware P FAU—Machira, Yvonne W., FAU MY, Yamo EF, Collymore YF, et al. Community perceptions of malaria and vaccines in the South Coast and Busia regions of Kenya. Malar. J. JID—101139802. doi: 10.1186/1475-2875-10-147 21624117 PMC3120733

[28] Febir LG, Asante KP, Dzorgbo DB, Senah KA, Letsa TS, Owusu-Agyei S. Community perceptions of a malaria vaccine in the Kintampo districts of Ghana. Malar. J. 2013 May 7;12: 156-2875-12-156. doi: 10.1186/1475-2875-12-156 23651533 PMC3656774

[29] Menaca A, Tagbor H, Adjei R, Bart-Plange C, Collymore Y, Ba-Nguz A, et al. Factors likely to affect community acceptance of a malaria vaccine in two districts of Ghana: a qualitative study. PLoS One 2014 Oct 15;9(10): e109707. doi: 10.1371/journal.pone.0109707 25334094 PMC4198134

[30] Cernuschi T, Gaglione S, Bozzani F. Challenges to sustainable immunization systems in GAVI transitioning countries. Vaccine. 2018;36(45): 6858–6866. doi: 10.1016/j.vaccine.2018.06.012 30268735 PMC6203805

[31] Ghana Districts. Akatsi North. 2017 [cited 2020 May 5] Available from: www.ghanadistricts.com/Home/District/173.

[32] Akatsi North District Assembly. Akatsi North: physical and natural environment. 2018 [cited 2020 May 11]. Available from: http://www.akatsinorth.gov.gh/economy.php.

[33] Ghana Health Service: Volta Regional Health Directorate. Volta Regional Health Directorate 2018 Annual Report. 2018.

[34] Geneau R, Massae P, Courtright P, Lewallen S. Using qualitative methods to understand the determinants of patients’ willingness to pay for cataract surgery: a study in Tanzania. Soc Sci Med 2008;66(3): 558–568. doi: 10.1016/j.socscimed.2007.09.016 18006201

[35] Ghana Statistical Service (GSS), Ghana Health Service (GHS) and ICF International. Ghana Demographic and Health Survey 2014. 2015.

[36] University Stanford. MacArthur Scale of Subjective Social Status–Adult Version. 2018 [cited 2019 Nov 30]. Available from: http://sparqtools.org/mobility-measure/macarthur-scale-of-subjective-social-status-adult-version/#all-survey-questions.

[37] Braun V, Clarke V. Using thematic analysis in psychology. Qual Res Psychol. 2006;3(2): 77–101.

[38] Saldaña J. The Coding Manual for Qualitative Researchers. 2nd ed.: SAGE; 2013.

[39] Barusch A, Gringeri C, George M. Rigor in Qualitative Social Work Research: A Review of Strategies Used in Published Articles. Soc Work Res. 2011;35(1): 11-12-19.

[40] GAVI. Ghana: Key information on co-financing. 2019 [cited 2020 Feb 18] Available from: https://www.gavi.org/sites/default/files/document/co-financing-information-sheet-ghanapdf.pdf.

[41] Ghana Statistical Service. Ghana living standards survey round 6. 2014;6.

[42] World Health Organization. Vaccine Pricing: GAVI Transitioning Countries. 2017 [cited 2020 Apr 17]. Available from: https://lnct.global/wp-content/uploads/2018/02/Vaccine-Pricing-for-GAVI-Transitioning-Countries-1.pdf.

[43] Republic of Angola, Immunization Multi-Year Plan. 2011 [cited 2020 May 1]. Available from: https://www.gavi.org/sites/default/files/country/cmyp/Angola-Comprehensive-multi-year-plan-for-2011-2015—Year-2011-.pdf.

[44] Ministry of Health, Royal Government of Bhutan. Bhutan Comprehensive Multi-Year Plan of Immunization. 2009–2013 [cited 2020 May 1]. Available from: https://www.gavi.org/sites/default/files/document/comprehensive-multi-year-plan-for-2009-2013pdf.pdf.

[45] Bhutan Health Trust Fund. BHTF Finance Accountability. 2020 [cited 2020 May 15]. Available at: https://www.bhtf.bt/finance-accountability.

[46] Market Express. Heaven Black Mosquito Coil. no date [cited 2020 May 14]. Available at: https://marketexpress.com.gh/heaven-black-mosquito-coil.

[47] Index Mundi. Ghana—PPP conversion factor. 2018 [cited 2020 May 7]. Available at: https://www.indexmundi.com/facts/ghana/ppp-conversion-factor.

[48] Hou Z, Chang J, Yue D, Fang H, Meng Q, Zhang Y. Determinants of willingness to pay for self-paid vaccines in China. Vaccine. 2014;32(35): 4471–4477. doi: 10.1016/j.vaccine.2014.06.047 24968160

[49] Worrall E, Basu S, Hanson K. Is malaria a disease of poverty? A review of the literature. Trop. Med. Int. Health. 2005;10(10): 1047–1059. doi: 10.1111/j.1365-3156.2005.01476.x 16185240

[50] Blanchet NJ, Fink G, Osei-Akoto I. The effect of Ghana’s National Health Insurance Scheme on health care utilisation. Ghana Med J 2012;46(2): 76–84. 22942455 PMC3426378

[51] Dimala CA, Kika BT, Kadia BM, Blencowe H. Current challenges and proposed solutions to the effective implementation of the RTS, S/AS01 Malaria Vaccine Program in sub-Saharan Africa: A systematic review. PLoS One 2018;13(12):e0209744. doi: 10.1371/journal.pone.0209744 30596732 PMC6312235

[52] Hogarh JN, Antwi-Agyei P, Obiri-Danso K. Application of mosquito repellent coils and associated self-reported health issues in Ghana. Malar. J. 2016;15(1): 61. doi: 10.1186/s12936-016-1126-8 26847206 PMC4743129

[53] Obala AA, Mangeni JN, Platt A, Aswa D, Abel L, Namae J, et al. What Is Threatening the Effectiveness of Insecticide-Treated Bednets? A Case-Control Study of Environmental, Behavioral, and Physical Factors Associated with Prevention Failure. PLoS One 2015 Jul 14;10(7):e0132778. doi: 10.1371/journal.pone.0132778 26171962 PMC4501815

[54] Singal AG, Higgins PD, Waljee AK. A primer on effectiveness and efficacy trials. Clin Transl Gastroenterol. 2014 Jan 2;5: e45. doi: 10.1038/ctg.2013.13 24384867 PMC3912314

[55] Pryce J, Richardson M, Lengeler C. Insecticide‐treated nets for preventing malaria. Cochrane Database Syst Rev. 2018(11). doi: 10.1002/14651858.CD000363.pub3 30398672 PMC6418392

[56] Moscibrodzki P, Dobelle M, Stone J, Kalumuna C, Chiu YM, Hennig N. Free versus purchased mosquito net ownership and use in Budondo sub-county, Uganda. Malar. J. 2018;17(1): 363. doi: 10.1186/s12936-018-2515-y 30326909 PMC6191916

[57] Meijer M, Verschuuren M, Weggen E. COVID-19 vaccines a global public good? Moving past the rhetoric and making work of sharing intellectual property rights, know-how and technology. Eur. J. Public Health. 2021;31(5):925–6. doi: 10.1093/eurpub/ckab144 34473255 PMC8499959

[58] National Health Service. Your baby’s vaccination and immunisation schedule. 2018 [cited 2020 May 17]. Available from: https://www.nhs.uk/start4life/baby/vaccinations-and-immunisations-baby/.

[59] Hinman AR, Orenstein WA, Rodewald L. Financing immunizations in the United States. Clin Infect Dis. 2004;38(10): 1440–1446. doi: 10.1086/420748 15156483

[60] Oxford Vaccine Group. Herd immunity. 2019 [cited 2020 May 1]. Available from: https://vk.ovg.ox.ac.uk/vk/herd-immunity.

[61] World Health Organization. Malaria. 2020 [cited 2020 May 1]. Available from: https://www.who.int/news-room/fact-sheets/detail/malaria.

[62] Dalaba MA, Akweongo P, Aborigo R, Awine T, Azongo DK, Asaana P, et al. Does the national health insurance scheme in Ghana reduce household cost of treating malaria in the Kassena-Nankana districts? Glob health action 2014;7(1): 23848. 24836443 10.3402/gha.v7.23848 PMC4021818

[63] GAVI. Annex B: Supplementary Contextual Analysis. 2018 [cited 2020 May 21]. Available from: https://www.gavi.org/sites/default/files/board/minutes/2018/28-nov/docs/11%20-%20Annex%20B%20-%20Supplementary%20contextual%20analyses.pdf.

