# Supplementary material for: “I would have to sell things in order to get the money”: A qualitative exploration of willingness to pay for the RTS,S/AS01 malaria vaccine in the Volta region, Ghana
Source: PLoS One. 2022 Jun 8;17(6):e0268009. doi: 10.1371/journal.pone.0268009 (PMC9176758; doi:10.1371/journal.pone.0268009)
Supplement: S1 Table — (DOCX) [file pone.0268009.s001.docx]

**S1 Table: Ghana EPI schedule**

Modified from World Health Organization [1]

**References**

[1] World Health Organization. Vaccination schedule for Ghana. 2022 [cited 2022 February 26]. Available from: <https://immunizationdata.who.int/pages/schedule-by-country/gha.html?DISEASECODE=&TARGETPOP_GENERAL=>.

| Vaccine description (short) | Vaccine description | Age administered | Total number of vaccines administered |
| --- | --- | --- | --- |
| BCG | Baccille Calmette Guérin vaccine | Birth | 1 |
| OPV | Oral polio vaccine | Birth, Weeks 6,10,14 | 4 |
| Rotavirus | Rotavirus vaccine | Weeks 6,10,14 | 3 |
| DTwP-Hib-HepB | Pentavalent vaccine (Diphtheria, Haemophilus Influenzae, Hepatitis B, Pertussis, Tetanus) | Weeks 6,10,14 | 3 |
| PCV-13 | Pneumococcal conjugate vaccine 13-valent | Weeks 6,10,14 | 3 |
| IPV | Inactivated polio vaccine | Weeks 14 | 1 |
| YF | Yellow fever vaccine | Month 9 | 1 |
| MR | Measles and rubella vaccine | Months 9,18 | 2 |
| Men_A | Meningococcal A conjugate vaccine | Month 18 | 1 |
|  |  |  | Total = 19 |
