# Supplementary material for: “I would have to sell things in order to get the money”: A qualitative exploration of willingness to pay for the RTS,S/AS01 malaria vaccine in the Volta region, Ghana
Source: PLoS One. 2022 Jun 8;17(6):e0268009. doi: 10.1371/journal.pone.0268009 (PMC9176758; doi:10.1371/journal.pone.0268009)
Supplement: S2 Table — (DOCX) [file pone.0268009.s002.docx]

**S2 Table: Topic guide summary**

| **Objective** | **Summary** |
| --- | --- |
| Introduction | - The research assistant introduced themselves and the principal investigator, then introduced the study goals and procedures in a language the participant could understand. - The participant had the opportunity to ask any questions. - Informed consent was given, and rapport built before interview commencement. |
| To explore participants’ awareness of routine vaccines and the new malaria vaccine | - Participants were asked about their knowledge and experiences of RTS,S compared to routine vaccines |
| To explore participants’ appreciation of routine vaccines and the new malaria vaccine for their child | - Participants were asked of their opinions of RTS,S compared to routine vaccines - Participants who had defaulted on the vaccination schedule in the past were probed for their experiences |
| To assess participants’ WTP for any routinely given childhood vaccine and the malaria vaccine | - Participants were probed for the average, maximum and minimum amounts that they would pay for 1. Routine vaccines, and 2. RTS,S. This was based on a scenario where vaccines are no longer provided for free. - Participants were asked to justify and compare the values that they had given. - If participants expressed an unwillingness to pay, they were also probed. - Before this set of questions, participants were reassured that there were no current plans to charge for the vaccine. |
| To explore the factors influencing the participant’s WTP partial or full cost for vaccines | - Participants were probed for factors which influenced the values that they gave. - Examples of probes were participant experiences, vaccine importance, their perception about vaccine safety and decision-making in their household. Demographics questions and previous responses were also used as prompts. - Participants were encouraged to think about sources of income to pay user fees for vaccinations. - Participants were encouraged to think about their acceptability of user fees should they be introduced. |
