# Supplementary material for: “I would have to sell things in order to get the money”: A qualitative exploration of willingness to pay for the RTS,S/AS01 malaria vaccine in the Volta region, Ghana
Source: PLoS One. 2022 Jun 8;17(6):e0268009. doi: 10.1371/journal.pone.0268009 (PMC9176758; doi:10.1371/journal.pone.0268009)
Supplement: S1 File — (DOCX) [file pone.0268009.s003.docx]

**S1 File: Demographics questionnaire and MacArthur Scale of Subjective Social Status**

(to be completed by the researcher)

Participant code____________________

Date of completion_________________

Completed by: Participant/Researcher
*(circle as appropriate)*

**Demographics questionnaire**

Participant’s name____________________________________________________

Participant’s contact________________________________________________________

Age_________________ Sex____________________________________

1. What is the highest education level you **completed**? (*circle as appropriate)*:
2. Never attended school **or did not complete primary school**
3. Primary school
4. Middle/Junior Secondary School/Junior High School
5. Secondary+
6. Employment status *(circle as appropriate)*:
7. Employed
8. Not currently employed but has worked in the last 12 months
9. Not currently employed and has not worked in the last 12 months
10. Marital status (*circle as appropriate)*:
11. Never married b) Married c) Living together

d) Divorced/separated e) Widowed

1. Religion______________________________________________________________
2. Monthly net income____________________________________________________
3. Place of residence (*circle as appropriate)*: Urban/Rural
4. Which of the following does your household own? (*circle as appropriate)*:
5. TV b) Radio c) Mobile telephone
6. Internet access e) Means of transport
7. In which of the following ways have you been exposed to information on malaria in the last 6 months?
8. TV b) Radio c) Newspaper d) Poster

e) Leaflet f) Health worker g) Community volunteer

1. Number of children_____________
2. Age of child(ren) offered the malaria vaccine________________
3. Sex of child(ren) offered the malaria vaccine________________
4. **Birth order** of child(ren) offered the malaria vaccine *(circle as appropriate)*
5. **1^st^ b) 2^nd^ c) 3^rd^ d) 4^th^ e) 5+**
6. Number of insecticide-treated nets per household____________
7. Indoor residual spraying within the last 12 months? (*circle as appropriate*): Yes/No
8. Are you registered with the national/district health insurance scheme (N/DHIS)? (*circle as appropriate*): Yes/No
9. Excluding childbirth, have you attended a health facility within the last 6 months? (*circle as appropriate*): Yes/No
10. MacArthur Scale of Subjective Social Status [36]

Participants were read the following scenario:


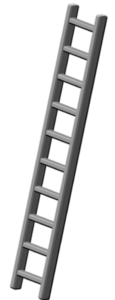
This ladder has 10 rungs. People at the top of the ladder are those who are wealthier, more educated, have the best jobs and are well off. People at the bottom of the ladder are least wealthy, least educated, the worst jobs or unemployed, and worse off. Which rung of the ladder best reflects where you think you fit?
